# Association of Smartphone Use With Body Image Distortion and Weight Loss Behaviors in Korean Adolescents

**DOI:** 10.1001/jamanetworkopen.2022.13237

**Published:** 2022-05-20

**Authors:** Sohyeon Kwon, Rockli Kim, Jong-Tae Lee, Jinho Kim, Sunmi Song, Seongcheol Kim, Hannah Oh

**Affiliations:** 1 Interdisciplinary Program in Precision Public Health, College of Health Science, Graduate School of Korea University, Seoul, Republic of Korea; 2 Department of Epidemiology, Johns Hopkins Bloomberg School of Public Health, Baltimore, Maryland; 3 Department of Health Policy and Management, College of Health Science, Korea University, Seoul, Republic of Korea; 4 Rehabilitation Science Program, Department of Health Science, Graduate School of Korea University, Seoul, Republic of Korea; 5 School of Media and Communication, Korea University, Seoul, Republic of Korea

## Abstract

**Importance:**

Despite high use of smartphones among adolescents, little is known about the association of smartphone use with body image and related behaviors.

**Objective:**

To examine the associations of duration of smartphone use and types of content most frequently accessed via smartphone with body image distortion and weight loss behaviors in adolescents.

**Design, Setting, and Participants:**

This cross-sectional study used data from the population-based Korea Youth Risk Behavior Web-Based Survey 2017. Participants comprised a nationally representative sample of 53 133 Korean adolescents aged 12 to 18 years. Data were collected from June 1 to July 18, 2017. The analysis was performed from February 7, 2020, to March 30, 2022.

**Exposures:**

Self-reported duration of smartphone use (min/d) and types of content (educational or informational searches; chatting, messaging, or email; social networking services or forums; games; videos, movies, or music; webtoons or web novels; and shopping or other activities) most frequently accessed during smartphone use.

**Main Outcomes and Measures:**

Body image distortion (overperception of body weight), weight loss attempt, use of inappropriate weight loss strategies (skipping meals, eating only 1 food at a time, vomiting, or using laxatives), and healthy weight loss behaviors (muscle-strengthening and aerobic physical activity). Multivariable logistic regression analysis was performed to estimate odds ratios (ORs) and 95% CIs, accounting for survey sampling and adjusting for potential confounders.

**Results:**

Among 53 133 participants, the mean (SD) age was 15.0 (1.8) years; 50.7% of participants were female, and 49.3% were male. After adjusting for types of content accessed, prolonged smartphone use (≥301 min/d) was positively associated with body image distortion (boys: OR, 1.17; 95% CI, 1.07-1.28; girls: OR, 1.20; 95% CI, 1.10-1.30) and inappropriate weight loss strategies (boys: OR, 1.54; 95% CI, 1.25-1.90; girls: OR, 2.45; 95% CI, 2.14-2.79) in both sexes compared with minimal smartphone use (1-120 min/d). After adjusting for duration of smartphone use, the use of smartphones mainly for videos, movies, or music (OR, 1.15; 95% CI, 1.02-1.29), webtoons or web novels (OR, 1.28; 95% CI, 1.10-1.48), and games (OR, 1.17; 95% CI, 1.03-1.32) was positively associated with body image distortion in boys compared with the use of smartphones mainly for educational or informational content. Among boys, the use of smartphones mainly for chatting, messaging, or email was positively associated with muscle-strengthening activity (OR, 1.31; 95% CI, 1.18-1.44) and aerobic physical activity (OR, 1.41; 95% CI, 1.29-1.55), as was the use of smartphones mainly for social networking services or forums (muscle-strengthening activity: OR, 1.27; 95% CI, 1.13-1.42; aerobic physical activity: OR, 1.28; 95% CI, 1.15-1.43). Among girls, the use of smartphones mainly for chatting, messaging, or email was positively associated with weight loss attempts (OR, 1.34; 95% CI, 1.19-1.51) and the use of inappropriate weight loss strategies (OR, 1.57; 95% CI, 1.25-1.99), as was the use of smartphones mainly for social networking services or forums (weight loss attempts: OR, 1.20; 95% CI, 1.07-1.36; use of inappropriate weight loss strategies: OR, 1.37; 95% CI, 1.08-1.73).

**Conclusions and Relevance:**

In this cross-sectional study, both the duration of smartphone use and the types of content most frequently accessed via smartphone were associated with body image distortion and weight loss behaviors in adolescents. These findings suggest a need for the identification of strategies to help adolescents develop healthy smartphone use behaviors.

## Key Points

**Question:**

Are the duration of smartphone use and types of content most frequently accessed via smartphone associated with body image distortion and weight loss behaviors among adolescents?

**Findings:**

In this cross-sectional study of 53 133 Korean adolescents, the duration of smartphone use and the types of content most frequently accessed via smartphone were independently associated with overperception of body weight and the use of inappropriate weight loss strategies.

**Meaning:**

This study’s findings suggest a need for the identification of strategies to help adolescents develop healthy smartphone use behaviors.

## Teaser

This cross-sectional study uses data from the Korea Youth Risk Behavior Web-Based Survey to examine the associations of duration of smartphone use and types of content most frequently accessed via smartphone with body image distortion and weight loss behaviors in Korean adolescents.

## Introduction

Over the past decade, the number of smartphone users has rapidly increased worldwide.^1^ Today, the smartphone has become a major screen device that is used to access various media content, including social media (eg, Instagram and Facebook), videos (eg, YouTube and Netflix), games, and music.^2^ Smartphones also conveniently allow rapid information searches and facilitate social interaction. Among smartphone users, adolescents have the longest duration of use^3^ and the highest risk of smartphone overdependence compared with any other age groups.^4^ Given the increased use of smartphones among adolescents, concerns have been raised regarding the negative consequences of smartphone use for adolescent development. Some studies have reported that excessive smartphone use is associated with depression,^5,6^ inadequate and low-quality sleep,^6^ and problems with social interactions^7^ among adolescents. Because adolescence is a sensitive period for psychological and behavioral development,^8,9^ smartphone use during adolescence is likely to be associated with mental health and related behaviors. However, little is known about the associations of smartphone use with other psychological outcomes and related health behaviors, including body image distortion (BID) and inappropriate weight loss attempts.

When using smartphones, adolescents are likely to be exposed to various content depicting images that idealize thinness.^10,11,12^ For example, fashion and beauty content that is popular among adolescents often attracts users by presenting photos and videos of models and celebrities, who are usually thinner than a typical person.^13^ Digital platforms may also prolong users’ exposure to such content via the use of web algorithms that constantly recommend and direct users to similar content.^14^ Continuous exposure to those images may create negative perceptions of one’s physical appearance, particularly overperception of body weight,^15,16^ and promote inappropriate weight loss attempts.^17,18,19,20^ Through social media and other networking platforms, adolescents may also interact with peers, spreading body dissatisfaction and the desire for an ideal body (eg, thinspiration [content idealizing and promoting thin bodies] or fitspiration [content idealizing and promoting strong, fit bodies]).^21^ Moreover, adolescents may obtain and share false information about weight loss strategies.^22^ Using inappropriate weight loss strategies, such as self-induced vomiting, use of laxatives or diuretics, and use of weight loss medications without prescription, rather than engaging in appropriate levels of physical activity may lead to nutritional deficiencies and growth deceleration.^23^ In many countries, eating disorders and inappropriate weight loss attempts have become a public health problem among adolescents.^24,25,26^

In this cross-sectional study, we examined the associations of smartphone use patterns (duration and content type) with BID (specifically overperception of body weight) and weight loss behaviors (weight loss attempts, use of inappropriate weight loss strategies, and healthy behaviors, including muscle-strengthening and aerobic physical activity) among adolescent smartphone users. While previous studies have primarily focused on smartphone addiction and problematic smartphone use,^27,28^ we examined a wider range of smartphone use duration and compared associations across multiple types of content accessed via smartphone by using nationally representative survey data from South Korea, a country with one of the highest smartphone penetration rates (>94% of adolescents own smartphones).^29^ Furthermore, because an individual’s susceptibility to BID and inappropriate weight loss behaviors as well as the specific content accessed using smartphones may vary by sex^30,31^ and socioeconomic status,^32^ we also assessed whether these variables modified any associations.

## Methods

This cross-sectional study was approved by the institutional review board of the Korea Disease Control and Prevention Agency. All participants provided written informed consent before beginning the survey. The study followed the Strengthening the Reporting of Observational Studies in Epidemiology (STROBE) reporting guideline for cross-sectional studies.^33^

### Study Population

The Korea Youth Health Risk Behavior Web-Based Online Survey (KYRBS) has been conducted annually since 2005 by the Korea Disease Control and Prevention Agency, the Ministry of Health and Welfare, and the Ministry of Education to monitor health behaviors in adolescents. Each year, a nationally representative sample of middle school and high school students (aged 12-18 years) is selected using a multistage cluster sampling design based on region, city size, and school type.^34^ Approximately 400 middle schools and 400 high schools participate in the survey each year. The survey is conducted via online self-administered questionnaires in the computer laboratory of each sampled school. The questionnaires collect information on sociodemographic characteristics, body measurements, mental health, and health behaviors, including weight loss attempts and physical activity.

The analysis was restricted to the KYRBS 2017 because it was the most recent version that collected information on both the duration of smartphone use and the types of content most frequently accessed via smartphone. Data were collected from June 1 to July 18, 2017. The analysis was performed from February 7, 2020, to March 30, 2022.

Among 62 276 adolescents who participated in the KYRBS 2017, those who were missing information on height and weight (n = 1884) were excluded. We further excluded 7259 participants who reported not using smartphones during the past 30 days because these adolescents may not have had access to smartphones and other resources required for practicing healthy lifestyles. The characteristics of participants who reported not using smartphones during the past 30 days are shown in eTable 1 in the Supplement. A total of 53 133 participants (26 220 male and 26 913 female) were included in the analysis.

### Assessment of Smartphone Use

During the survey, participants were asked to report their mean weekday and weekend duration of smartphone use (min/d) during the past 30 days. Using the self-reported data on weekday and weekend use, we estimated the mean daily duration of smartphone use (calculated as the sum of 5 times the weekday duration and 2 times the weekend duration, then divided by 7) and categorized this use into quartiles (1-120 min/d, 121-180 min/d, 181-300 min/d, and ≥301 min/d). Participants also reported the single most frequently accessed content among 13 types: educational (eg, online lectures), informational searches, chatting or messaging, games, movies, webtoons (ie, digital comics) or web novels, music, videos (eg, YouTube), social networking services (eg, blogs, Instagram, Twitter, and Facebook), online community forums, email, online shopping, and other activities. Based on the similarities, we collapsed the content types into 7 groups: educational or informational searches; chatting, messaging, or email; social networking services or forums; games; videos, movies, or music; webtoons or web novels; and shopping or other activities.

### Assessment of Body Image Distortion

Body weight and height were self-reported via online survey. Based on the 2017 Korean National Growth Chart,^35,36^ underweight was defined as having a body mass index (BMI; calculated as weight in kilograms divided by height in meters squared) lower than the 5th percentile for age and sex, normal weight as having a BMI between the 5th and 84th percentile for age and sex, and overweight as having a BMI in the 85th percentile or higher for age and sex. Participants reported their perceptions of their current body weight as very lean, lean, normal weight, fat, or very fat; the words fat and very fat were chosen because the survey measured respondents’ perceptions, and these words are commonly used by adolescents. In our analyses, we defined BID as overperception of body weight, with participants classified as having BID if they were (1) underweight but perceived themselves as normal weight, fat, or very fat; (2) normal weight but perceived themselves as fat or very fat; or (3) overweight but perceived themselves as very fat. In sensitivity analyses, we further divided the categories into underperception, accurate perception, and overperception of body weight and examined the associations of smartphone use patterns with both underperception and overperception of body weight.

### Assessment of Weight Loss Behaviors

Participants were asked to report whether they tried to lose weight during the past 30 days and whether they used any of the following inappropriate weight loss strategies: skipping meals for more than 24 hours, eating only 1 food item, inducing vomiting after a meal, using laxatives or diuretics, and using weight loss medications without prescription. Healthy behaviors included aerobic and muscle-strengthening activity. For aerobic physical activity, participants reported the number of days they engaged in at least 60 minutes of moderate- to vigorous-intensity physical activity and the number of days they engaged in at least 20 minutes of vigorous-intensity physical activity during the past 7 days. For muscle-strengthening activity (eg, doing pushups or lifting weights), participants reported the number of days they performed that activity during the past 7 days. For both muscle-strengthening and aerobic physical activity, we dichotomized participants into 2 groups based on the national physical activity guidelines for adolescents (moderate- to vigorous-intensity aerobic physical activity at least 5 days/week or vigorous-intensity aerobic activity at least 3 days/week; muscle-strengthening activity at least 3 days/week).^37^

### Statistical Analysis

All statistical analyses accounted for sampling weights and the complex sampling design of the survey. Study participants were weighted to represent the entire Korean population of middle school and high school students. We performed weighted multivariable logistic regression analysis to estimate odds ratios (ORs) and 95% CIs for the associations of smartphone use patterns (duration and content type) with BID and weight loss behaviors. Multivariable models were adjusted for potential confounders, including grade in school, household income, parental coresidence, parental educational level, region, perceived stress level, presence of depressive symptoms, and BMI. In all models, we mutually adjusted for the duration of smartphone use and the type of content most frequently accessed to examine the independent association of each variable. For the duration analysis, we tested for trend using the Wald test for category-specific medians.^38^ For the content analysis, we tested for overall difference among the categories using a global *F* test.^39^

In secondary analyses, we examined the role of BID in the association between smartphone use patterns and weight loss behaviors by comparing models with and without additional adjustment for BID. Because adolescents who already had BID (one of the most important risk factors for weight loss behaviors^18,20^) may have been more susceptible to the consequences of smartphone use, we also examined the association of smartphone use patterns with weight loss behaviors after stratification by BID. To examine whether the association with duration of smartphone use varied by the type of content most frequently accessed, we also stratified the duration analysis by content type and tested for interaction. In addition, we stratified the analyses by sex and socioeconomic factors (household income and parental educational level), which were potential modifiers in the associations. We tested for interaction using the Wald test for product terms (ie, smartphone use patterns and the variable being evaluated). Because most interactions by sex were statistically significant, we reported the results in male and female adolescents separately.

In sensitivity analyses, we conducted separate analyses for weekday and weekend smartphone use to examine the variation in associations by day of smartphone use. Because overperception of body weight among adolescents who are overweight may be more difficult to define and subject to greater measurement error (eg, the ceiling effect), we also repeated the analyses after excluding those adolescents. In separate sensitivity analyses, we stratified our analyses by BMI (underweight, normal weight, and overweight) and further investigated the association of smartphone use duration with both underperception and overperception of body weight using multivariable polytomous logistic regression models, with accurate perception as the reference outcome. To evaluate the consequences of recent changes in smartphone use patterns and the digital environment, we examined associations with smartphone use duration using more recent data from the KYRBS 2020. Because the KYRBS 2020 collected information on smartphone use duration but not content types, we used these data in sensitivity analyses only.

All statistical tests were 2-sided with a significance threshold of α = .05. We also adjusted for multiple testing using Bonferroni correction (5 tests per exposure). All analyses were performed using SURVEY procedures in SAS software, version 9.4 (SAS Institute Inc).

## Results

### Participant Characteristics

Among 53 133 participants, the mean (SD) age at the time of survey completion was 15.0 (1.8) years; 50.7% of participants were female, and 49.3% were male. Among male adolescents, the weighted prevalence of BID was 19.8%, and the use of inappropriate weight loss strategies in the past 30 days was 2.9%. Among female adolescents, the weighted prevalence of BID was 31.6%, and the use of inappropriate weight loss strategies in the past 30 days was 9.4%. The mean (SE) duration of smartphone use was 212.7 (2.2) minutes per day in boys and 263.8 (2.9) minutes per day in girls. The duration of smartphone use was longest among participants who used smartphones mainly for social networking services or forums (mean [SE], 280.9 [2.8] min/d) and shortest among participants who used smartphones mainly for educational or informational searches (mean [SE], 158.3 [3.1] min/d) (eTable 2 in the Supplement). Additional participant characteristics by duration of smartphone use are shown in Table 1. Adolescents with prolonged smartphone use (≥301 min/d) vs minimal smartphone use (1-120 min/d) were more likely to be female (60.4% vs 37.9%), live in rural areas (7.5% vs 5.6%) or small- to medium-sized urban areas (53.8% vs 46.9%), live with a single parent (18.3% vs 10.0%), and have low household income (19.0% vs 9.8%), low parental educational levels (eg, middle school or lower: 1.3% vs 0.5%), high levels of stress (44.0% vs 32.8%), and depressive symptoms (32.2% vs 20.4%).

**Table 1.** Participant Characteristics by Duration of Smartphone Use (N = 53 133)

Characteristic	Participants, unweighted No. (weighted %)^a^			
	Duration of smartphone use			
	1-120 min/d	121-180 min/d	181-300 min/d	≥301 min/d
Total participants, No.				
Unweighted	14 092	11 144	15 043	12 854
Weighted	698 144	551 804	730 726	605 491
School^b^				
Middle school	6994 (45.8)	5358 (43.8)	7462 (45.2)	6527 (46.3)
High school	7098 (54.2)	5786 (56.2)	7581 (54.8)	6327 (53.7)
Sex				
Male	8641 (62.1)	5911 (54.4)	6746 (45.9)	4922 (39.6)
Female	5451 (37.9)	5233 (45.6)	8297 (54.1)	7932 (60.4)
Household income				
Low	1402 (9.8)	1335 (11.8)	2219 (14.6)	2450 (19.0)
Middle	5802 (40.6)	5256 (46.9)	7395 (49.1)	6289 (48.8)
High	6888 (49.6)	4553 (41.3)	5429 (36.4)	4115 (32.2)
Parental coresidence				
Both parents	12 325 (88.1)	9585 (86.8)	12 504 (83.7)	9997 (78.4)
Single parent	1455 (10.0)	1319 (11.4)	2167 (14.0)	2405 (18.3)
Not living with parents	312 (1.9)	240 (1.8)	372 (2.3)	452 (3.3)
Parental educational level^c^				
≤Middle school	83 (0.5)	79 (0.7)	152 (0.9)	186 (1.3)
High school	2408 (16.8)	2477 (22.2)	4050 (26.6)	3986 (30.7)
≥College	10 071 (73.0)	7190 (65.4)	8736 (59.2)	6267 (50.2)
Missing	1530 (9.7)	1398 (11.7)	2105 (13.3)	2415 (17.8)
Region				
Rural	946 (5.6)	717 (5.0)	1159 (6.0)	1221 (7.5)
Small- and medium-sized urban	6274 (46.9)	5296 (50.5)	7277 (51.5)	6471 (53.8)
Metropolitan urban	6872 (47.6)	5131 (44.6)	6607 (42.5)	5162 (38.8)
Perceived stress level				
Low	3432 (23.7)	2336 (20.4)	2799 (18.5)	2038 (16.0)
Moderate	6065 (43.5)	5011 (45.5)	6470 (43.1)	5094 (40.0)
High	4595 (32.8)	3797 (34.1)	5774 (38.4)	5722 (44.0)
Presence of depressive symptoms	2857 (20.4)	2492 (22.5)	3851 (25.7)	4138 (32.2)
Content types of smartphone use				
Educational or informational searches	2081 (15.5)	807 (7.8)	765 (5.4)	411 (3.5)
Chatting, messaging, or emails	3359 (24.0)	3038 (27.7)	4124 (27.6)	4123 (32.2)
Social networking services or forums	1809 (12.7)	2032 (17.7)	3325 (21.7)	3362 (25.8)
Videos, movies, or music	3000 (21.3)	2569 (23.3)	3463 (23.4)	2452 (19.2)
Webtoons or web novels	1441 (10.2)	1055 (9.4)	1364 (8.9)	919 (7.1)
Games	2240 (15.3)	1556 (13.5)	1867 (12.0)	1418 (10.9)
Shopping or other activities	162 (1.0)	87 (0.7)	135 (0.9)	169 (1.2)
BMI^d^				
Underweight	982 (7.1)	743 (6.5)	1054 (7.0)	863 (6.9)
Normal weight	10 549 (74.8)	8385 (75.6)	11 099 (73.9)	9272 (72.1)
Overweight	2561 (18.1)	2016 (17.9)	2890 (19.1)	2719 (21.0)
Body image distortion^e^	3116 (22.2)	2703 (24.6)	4067 (27.0)	3731 (28.8)
Weight loss attempt	3837 (27.0)	3444 (30.5)	5277 (34.9)	5061 (39.0)
Use of inappropriate weight loss strategies	511 (3.5)	526 (4.6)	945 (6.2)	1382 (10.5)
Muscle-strengthening activity that met guidelines	3570 (25.0)	2592 (22.9)	3211 (21.2)	2672 (20.6)
Aerobic physical activity that met guidelines	6147 (42.2)	4513 (39.3)	5639 (36.4)	4688 (35.7)

Abbreviation: BMI, body mass index (calculated as weight in kilograms divided by height in meters squared).

^a^ Percentages were weighted to the entire population of middle-school and high school students in Korea.

^b^ Grade in school was used as a proxy for age in the analyses.

^c^ Organization for Economic Co-operation and Development (OECD) parental education indicators: middle school or lower indicates both parents completed middle school education or less; high school indicates at least 1 parent graduated from high school; college or higher indicates at least 1 parent graduated from college or more.

^d^ Underweight was defined as BMI for age that was lower than the 5th percentile, normal weight as BMI for age that was between the 5th and 84th percentile, and overweight as BMI for age that was in the 85th percentile or higher based on the 2017 Korean National Growth Chart.

^e^ Body image distortion was defined as overperception of body weight, which occurred when participants who were underweight perceived themselves as normal weight, fat, or very fat; when participants who were normal weight perceived themselves as fat or very fat; and when participants with overweight perceived themselves as very fat.

### Duration of Smartphone Use

After adjusting for content type, prolonged smartphone use (≥301 min/d) was associated with a higher prevalence of BID in both boys (OR, 1.17; 95% CI, 1.07-1.28; *P* = .001 for trend) and girls (OR, 1.20; 95% CI, 1.10-1.30; *P* < .001 for trend) compared with minimal smartphone use (1-120 min/d) (Table 2). Prolonged smartphone use was also positively associated with the use of inappropriate weight loss strategies in both boys (OR, 1.54; 95% CI, 1.25-1.90; *P* < .001 for trend) and girls (OR, 2.45; 95% CI, 2.14-2.79; *P* < .001 for trend). Among female adolescents, prolonged smartphone use was also positively associated with weight loss attempts (OR, 1.57; 95% CI, 1.45-1.69; *P* < .001 for trend). The extent of associations was higher among female vs male adolescents for weight loss attempts (OR, 1.57 vs 1.06; *P* < .001 for interaction) and the use of inappropriate weight loss strategies (OR, 2.45 vs 1.54; *P* < .001 for interaction), with a statistically significant interaction by sex. The association between duration of smartphone use and weight loss behaviors did not change after additional adjustment for BID (eTable 3 in the Supplement).

**Table 2.** Association of Duration of Smartphone Use With Body Image Distortion and Weight Loss Behaviors in Male and Female Adolescents

Outcome	Sex	Events, No.	OR (95% CI)^a^				*P* for trend^b^	*P* for interaction by sex^c^
			Duration of smartphone use					
			1-120 min/d	121-180 min/d	181-300 min/d	≥301 min/d		
Body image distortion^d^	Male	5205	1 [Reference]	1.07 (0.99-1.16)	1.13 (1.05-1.23)	1.17 (1.07-1.28)	.001^e^	.40
	Female	8412	1 [Reference]	1.06 (0.98-1.15)	1.15 (1.06-1.24)	1.20 (1.10-1.30)	<.001^e^	
Weight loss attempt	Male	6174	1 [Reference]	1.02 (0.94-1.11)	1.15 (1.07-1.24)	1.06 (0.97-1.16)	.09	<.001^e^
	Female	11 445	1 [Reference]	1.20 (1.11-1.29)	1.35 (1.26-1.45)	1.57 (1.45-1.69)	<.001^e^	
Use of inappropriate weight loss strategies	Male	782	1 [Reference]	1.08 (0.86-1.36)	1.18 (0.97-1.44)	1.54 (1.25-1.90)	<.001^e^	<.001 ^e^
	Female	2582	1 [Reference]	1.23 (1.06-1.42)	1.53 (1.34-1.76)	2.45 (2.14-2.79)	<.001^e^	
Muscle-strengthening activity^f^	Male	9020	1 [Reference]	0.99 (0.92-1.07)	1.04 (0.97-1.11)	1.03 (0.96-1.11)	.30	.68
	Female	3025	1 [Reference]	0.95 (0.84-1.08)	0.94 (0.84-1.05)	1.04 (0.92-1.18)	.24	
Aerobic physical activity^g^	Male	13 870	1 [Reference]	1.03 (0.96-1.10)	1.01 (0.95-1.07)	1.04 (0.96-1.12)	.41	.97
	Female	7117	1 [Reference]	0.96 (0.88-1.06)	0.95 (0.87-1.04)	1.02 (0.93-1.11)	.39	

Abbreviation: OR, odds ratio.

^a^ Adjusted for grade in school (continuous), sex (male or female), household income (low, middle, or high), parental coresidence (both parents, single parent, or not living with parents or others), parental educational level (middle school or less, high school, college or more, or missing), region (rural, small and medium-sized urban, or metropolitan urban), perceived stress level (low, moderate, or high), depressive symptoms (yes or no), body mass index (underweight, normal weight, or overweight), and type of content most frequently accessed during smartphone use (educational or informational searches; chatting, messaging, or emails; games; videos, movies, or music; webtoons or web novels; social networking services or forums; or shopping or other activities).

^b^ *P* for trend was estimated using the Wald test for continuous duration of smartphone use (with category-specific medians).

^c^ *P* for interaction by sex was estimated using the Wald test for the interaction term between sex and duration of smartphone use (with category-specific medians).

^d^ Body image distortion was defined as overperception of body weight, which occurred when participants who were underweight perceived themselves as normal weight, fat, or very fat; when participants who were normal weight perceived themselves as fat or very fat; and when participants with overweight perceived themselves as very fat.

^e^ Nominal statistical significance at Bonferroni-corrected α = .01 (5 tests per exposure).

^f^ Defined as engagement in muscle-strengthening activity for at least 3 days per week.

^g^ Defined as engagement in moderate- to vigorous-intensity aerobic physical activity at least 5 days per week or vigorous-intensity physical activity at least 3 days per week.

When stratified by BID, similar associations were observed among participants with and without BID (eTable 4 in the Supplement). Similar associations with duration of smartphone use were observed across all content types (eTable 5 in the Supplement). When we stratified by BMI and examined the associations with both underperception and overperception of body weight, the positive associations were restricted to overperception (eg, male adolescents with overweight with smartphone use ≥301 min/d vs 1-120 min/d: OR, 1.44; 95% CI, 1.20-1.73; *P* < .001 for trend), with the exception of male adolescents with normal weight, in which a positive association with underperception was also observed (eg, ≥301 min/d vs 1-120 min/d: OR, 1.18; 95% CI, 1.07-1.30; *P* = .002 for trend) (eTable 6 in the Supplement).

### Content Types of Smartphone Use

Among male adolescents, using smartphones most frequently for videos, movies, or music (OR, 1.15; 95% CI, 1.02-1.29), webtoons or web novels (OR, 1.28; 95% CI, 1.10-1.48), and games (OR, 1.17; 95% CI, 1.03-1.32) compared with educational or informational searches was associated with a higher prevalence of BID after adjustment for total duration of smartphone use (*P* < .001) (Table 3). Among male adolescents, higher levels of muscle-strengthening and aerobic physical activity were associated with the use of smartphones mainly for chatting, messaging, or email (muscle-strengthening activity: OR, 1.31; 95% CI, 1.18-1.44; aerobic activity: OR, 1.41; 95% CI, 1.29-1.55) and social networking services or forums (muscle-strengthening activity: OR, 1.27; 95% CI, 1.13-1.42; aerobic activity: OR, 1.28; 95% CI, 1.15-1.43). Among female adolescents, the use of smartphones mainly for webtoons or web novels was positively associated with BID prevalence (OR, 1.15; 95% CI, 1.00-1.33), and the use of smartphones mainly for chatting, messaging, or email (OR, 1.34; 95% CI, 1.19-1.51) and social networking services or forums (OR, 1.20; 95% CI, 1.07-1.36) was positively associated with weight loss attempts. Inappropriate weight loss strategies were also associated with the use of smartphones mainly for chatting, messaging, or email (OR, 1.57; 95% CI, 1.25-1.99) and social networking services or forums (OR, 1.37; 95% CI, 1.08-1.73) among female adolescents. Among both male and female adolescents, the use of smartphones mainly for games was inversely associated with aerobic physical activity (boys: OR, 0.87; 95% CI, 0.79-0.95; girls: OR, 0.72; 95% CI, 0.59-0.89). All interactions by sex were statistically significant. The associations with weight loss behaviors did not change after additional adjustment for BID (eTable 7 in the Supplement).

**Table 3.** Association of Smartphone Content With Body Image Distortion and Weight Loss Behaviors in Male and Female Adolescents

Outcome	Sex	Events, No.	OR (95% CI)^a^							*P* value ^b^	*P* for interaction by sex ^c^
			Content type								
			Educational or informational searches	Chatting, messaging, or emails	Social networking services or forums	Videos, movies, or music	Webtoons or web novels	Games	Shopping or other activities		
Body image distortion^d^	Male	5205	1 [Reference]	0.94 (0.83-1.06)	0.87 (0.75-1.00)	1.15 (1.02-1.29)	1.28 (1.10-1.48)	1.17 (1.03-1.32)	1.04 (0.75-1.44)	<.001^e^	.001^e^
	Female	8412	1 [Reference]	0.98 (0.86-1.11)	1.00 (0.88-1.14)	1.11 (0.97-1.26)	1.15 (1.00-1.33)	1.05 (0.87-1.26)	1.03 (0.78-1.36)	.003^e^	
Weight loss attempt	Male	6174	1 [Reference]	1.10 (0.97-1.24)	1.13 (0.99-1.29)	1.07 (0.95-1.20)	0.90 (0.78-1.05)	0.94 (0.84-1.06)	0.82 (0.59-1.15)	.001^e^	<.001^e^
	Female	11 445	1 [Reference]	1.34 (1.19-1.51)	1.20 (1.07-1.36)	1.09 (0.96-1.23)	0.88 (0.77-1.01)	0.71 (0.60-0.85)	1.26 (0.99-1.61)	<.001^e^	
Use of inappropriate weight loss strategies	Male	782	1 [Reference]	1.04 (0.75-1.44)	1.18 (0.84-1.67)	0.98 (0.72-1.34)	0.77 (0.53-1.12)	0.89 (0.64-1.24)	1.00 (0.49-2.05)	.13	.04
	Female	2582	1 [Reference]	1.57 (1.25-1.99)	1.37 (1.08-1.73)	1.14 (0.89-1.46)	0.93 (0.71-1.22)	0.93 (0.65-1.33)	1.37 (0.90-2.07)	<.001^e^	
Muscle-strengthening activity^f^	Male	9020	1 [Reference]	1.31 (1.18-1.44)	1.27 (1.13-1.42)	0.95 (0.86-1.04)	0.96 (0.85-1.08)	0.82 (0.74-0.91)	1.41 (1.08-1.85)	<.001^e^	<.001^e^
	Female	3025	1 [Reference]	1.08 (0.89-1.31)	0.96 (0.79-1.17)	1.10 (0.91-1.32)	0.81 (0.65-1.01)	0.77 (0.58-1.01)	1.00 (0.68-1.47)	<.001^e^	
Aerobic physical activity^g^	Male	13 870	1 [Reference]	1.41 (1.29-1.55)	1.28 (1.15-1.43)	1.01 (0.92-1.11)	0.96 (0.85-1.08)	0.87 (0.79-0.95)	1.82 (1.40-2.36)	<.001^e^	<.001^e^
	Female	7117	1 [Reference]	0.95 (0.84-1.08)	0.85 (0.74-0.97)	0.97 (0.84-1.11)	0.90 (0.78-1.04)	0.72 (0.59-0.89)	1.05 (0.78-1.41)	<.001^e^	

Abbreviation: OR, odds ratio.

^a^ Adjusted for grade in school (continuous), sex (male or female), household income (low, middle, or high), parental coresidence (both parents, single parent, or not living with parents or others), parental education (middle school or less, high school, college or more, or missing), region (rural, small and medium-sized urban, or metropolitan urban), perceived stress level (low, moderate, or high), depressive symptoms (yes or no), body mass index (underweight, normal weight, or overweight), and duration of smartphone use (≤120 min/d, 121-180 min/d, 181-300 min/d, or ≥301 min/d).

^b^ *P* value was estimated using a global *F* test comparing across types of content most frequently accessed during smartphone use.

^c^ *P* for interaction by sex was estimated using a global *F* test for interaction terms between sex and types of content accessed during smartphone use.

^d^ Body image distortion was defined as overperception of body weight, which occurred when participants who were underweight perceived themselves as normal weight, fat, or very fat; when participants who were normal weight perceived themselves as fat or very fat; and when participants with overweight perceived themselves as very fat.

^e^ Nominal statistical significance at Bonferroni-corrected α = .01 (5 tests per exposure).

^f^ Defined as engagement in muscle-strengthening activity for at least 3 days per week.

^g^ Defined as engagement in moderate- to vigorous-intensity aerobic physical activity at least 5 days per week or vigorous-intensity physical activity at least 3 days per week.

Most associations remained statistically significant after Bonferroni correction for multiple testing (Table 2 and Table 3). In analyses stratified by household income and parental educational level, the direction of associations was largely consistent in all subgroups (eTable 8 in the Supplement). Similar associations were observed for the duration of smartphone use on weekdays and weekends (eTable 9 in the Supplement), after excluding adolescents with overweight (eTable 10 and eTable 11 in the Supplement), and after using more recent data from the KYRBS 2020 (eTable 12 in the Supplement).

## Discussion

In this cross-sectional study of a nationally representative sample of Korean adolescents, we found that prolonged smartphone use was associated with a higher prevalence of BID and the use of inappropriate weight loss strategies. With adjustment for duration of smartphone use, the use of smartphones mainly for entertainment-focused content (such as videos, movies, music, webtoons, web novels, and games) was associated with a higher prevalence of BID. Using smartphones mainly for interaction-focused content (such as chatting, messaging, email, social networking services, and forums) was associated with higher levels of muscle-strengthening and aerobic physical activity in male adolescents. In female adolescents, using smartphones mainly for interaction-focused content was positively associated with weight loss attempts and the use of inappropriate weight loss strategies. These data suggest that both duration of smartphone use and types of content accessed via smartphone were independently associated with BID and weight loss behaviors among adolescents.

Our findings of a positive association between prolonged smartphone use and BID are consistent with results from previous studies on internet addiction.^40,41,42^ In those studies, adolescents reporting internet addiction symptoms were more likely to experience overperception of body weight and body image avoidance (ie, behavioral avoidance of situations in which one’s body shape is conspicuous).^40,41,42^ In the present study, we observed a positive association with BID among adolescents with moderate ranges of smartphone use duration that are commonly found in adolescents without addiction problems, suggesting that even moderate use of smartphones may have unhealthy consequences for body image. Furthermore, these associations persisted after adjusting for content types, supporting the independent associations with duration of smartphone use. Individuals with prolonged smartphone use are more likely to be exposed to idealized body images and appearance-related content from various digital platforms (eg, social media, blog posts, and advertisements),^14^ which provide increased opportunities for cognitive internalization of idealized body shapes and engagement in body comparison that may lead to negative body image.^43^ The perception of body weight may also be associated with misleading messages and content from marketing companies^11^ and peers on digital platforms. Marketing companies, including those advertising weight loss products, often feature thin models and celebrities to promote their products, delivering the message that if you use their products, you too can become thin and beautiful. Unsubstantiated claims and deceptive advertising are particularly prevalent in online advertisements of weight loss products.^44,45^ Moreover, personalized web algorithms may lead users to consume more extreme content that triggers unhealthy perceptions and behaviors.^46^

In the present study, we also observed that, given a similar total duration of smartphone use, male and female adolescents who used smartphones most frequently for entertainment-focused content (such as videos, movies, music, webtoons, web novels, and games) had a higher BID prevalence compared with those who used smartphones to access other content. Other studies^47,48,49,50^ have also reported that children and adolescents who watched music videos^48,49,50^ and played appearance-focused internet games, such as dress-up or makeover games (eg, those in which players change the clothes and hairstyle of a female character to match the male character’s dream date),^47^ were more likely to experience body dissatisfaction. Many modern online games that are easily accessible via smartphone are focused on appearance, depicting images of authentic-looking game characters (or avatars) with unrealistic idealized body shapes.^51^ Those who listen to music on smartphones are also more likely to be exposed to music videos that feature thin models and celebrities (eg, K-pop stars).^48,49,50^ Visual exposure to images idealizing thinness may intensify internalization of this thin ideal and trigger overperception of one’s body weight. While these previous studies primarily focused on investigating the associations of single specific types of media content,^15,49,50,52^ our study compared the associations among different content types and found that exposure to entertainment-focused content may be associated with a more negative body image than other content types. Adolescents’ cognitive internalization may be more sensitive and responsive to visual exposure.^53^ In addition, visual memory and imagery have been identified as important factors associated with body dissatisfaction in young women.^54^

Our findings also suggest that the associations with smartphone use are not limited to negative body image and may further extend to weight loss behaviors. In the present study, prolonged smartphone use was associated with more prevalent use of inappropriate weight loss strategies among both male and female adolescents. Previous studies also reported a higher prevalence of eating disorders and laxative intake associated with prolonged internet use^55^ and internet addiction^41^ in adolescents. These associations are consistent with the positive association we observed with BID, an important risk factor for weight loss behaviors,^18,20^ suggesting that BID may be a possible link in the association between smartphone use and weight loss behaviors. However, we also observed that the positive association between prolonged smartphone use and inappropriate weight loss strategies was not attenuated after adjustment for BID, suggesting that BID-independent mechanisms may exist. Through digital platforms, adolescents may access and share diverse information about health, beauty, and weight management that have not been validated. Adolescents may be exposed to dramatic advertisements of weight loss products and anecdotes of extreme weight loss strategies.^44,45^ Adolescents may also easily create and spread unhealthy ideas and information to peers. Digital platforms also provide opportunities for adolescents to learn behaviors from peers who attract attention (eg, those receiving many likes on social media) and gather information about normative behaviors that are common and accepted among peers. Digital media, particularly given its ease of accessibility, may act as a super peer that substantially impacts adolescents’ perceptions and behaviors.^56,57^ Studies^58,59,60^ have found that digital media may have implications for adolescents’ risky behaviors, including smoking and substance use, through mechanisms associated with social norms that are created by the content and ideas shown and discussed in digital platforms.

Furthermore, using smartphones mainly for interaction-focused content, such as social networking services, forums, chatting, and messaging, was more closely associated with weight loss behaviors compared with other content types. Our data suggest that, in terms of behavioral change, communicating ideas and sharing tips about weight loss strategies with peers or like-minded individuals on social networking platforms may be more relevant than mere exposure to visual images of distant figures (eg, celebrities or game characters). In the present study, we also observed some variation in associations by sex. Interaction-focused content was associated with higher levels of physical activity in male adolescents; however, in female adolescents, interaction-focused content was associated with a higher prevalence of inappropriate weight loss attempts and strategies. The discrepant findings by sex may be partially explained by different targeting of thinspiration vs fitspiration^61^ messages and imagery between the sexes. A previous study reported that women were more likely to encounter thinspiration, and men more likely to encounter fitspiration, messages and imagery.^31^ Digital content and web algorithms are likely designed to target each sex differently, reflecting gender characteristics and stereotypes.^62^ Our finding of higher levels of physical activity associated with interaction-focused content among male adolescents also suggests that smartphone use may have some association with healthy adolescent behaviors if appropriate messages are delivered. Further investigation may be needed to clarify the role of smartphone use in weight loss behaviors among male vs female adolescents.

Compared with other content types, the use of smartphones to play games was also associated with lower levels of physical activity in both sexes. A previous study also reported lower levels of physical activity among male adolescents who played computer games.^63^ The associations may be partially explained by the higher likelihood of sedentary lifestyles among individuals who regularly play games on smartphones and computers.^64^ Further studies are needed to clarify the mechanisms associated with different smartphone content and weight loss behaviors.

### Strengths and Limitations

This study has several strengths. First, by using nationally representative data, we increased the generalizability of our results. Second, the use of a large sample allowed for the performance of various stratified analyses with sufficient statistical power. Third, we reduced confounding through careful adjustment for potential confounders, including socioeconomic status and psychological factors. We also examined the independent associations of smartphone use duration and content type by mutually adjusting within the models.

This study also has limitations. First, given the cross-sectional design, it is difficult to clarify the temporal association between exposure and outcome variables. Further studies are needed to confirm our results using a longitudinal design. Second, we used self-reported data on body weight and smartphone use, which are subject to measurement errors. Measurement errors of body weight may have led to misclassification of BID. Although the questionnaires on self-perception of body weight and weight loss behaviors have previously been found to have good reliability in both sexes,^65^ the validity and reliability of other variables, such as smartphone use patterns, have not been evaluated. Furthermore, when assessing smartphone content, participants were asked to report the single most frequently accessed type of content. Therefore, the associations we observed for specific content types may reflect the mixed effects of several content types if participants simultaneously used multiple types.

## Conclusions

This cross-sectional study found that both the duration of smartphone use and the types of content accessed via smartphone were associated with overperception of body weight and the use of inappropriate weight loss strategies among adolescents. Because accessibility to various digital content via smartphone has become easier, the inclusion of digital literacy education in health promotion strategies is needed to help adolescents critically evaluate and interpret the messages they encounter on digital platforms. At home, appropriate guidance is warranted to help adolescents develop healthy smartphone use behaviors. Digital platform providers may also consider taking more social responsibility for the content created and distributed on their platforms, thereby increasing their positive influence.

## Notes

Supplement. **eTable 1.** Participant Characteristics According Smartphone Use Duration, Including Adolescents Who Reported No Smartphone Use During the Past 30 Days **eTable 2.** Distribution of Smartphone Use Duration (Minutes/Day) According to Individuals’ Most Frequently Used Smartphone Content Type in Male and Female Adolescents **eTable 3.** Association of Smartphone Use Duration With Body Image Distortion (Overperception of Body Weight) and Weight Loss Behaviors (Weight Loss Attempts, Use of Inappropriate Weight Loss Strategies, Muscle-Strengthening Activity, and Aerobic Physical Activity) With Additional Adjustment for Body Image Distortion Among Male and Female Adolescents **eTable 4.** Association of Smartphone Use Duration With Weight Loss Behaviors (Weight Loss Attempts, Use of Inappropriate Weight Loss Strategies, Muscle-Strengthening Activity, and Aerobic Physical Activity) in Male and Female Adolescents, Stratified by Body Image Distortion **eTable 5.** Association of Smartphone Use Duration With Body Image Distortion and Weight Loss Behaviors Among Male and Female Adolescents, Stratified by Types of Content Most Frequently Accessed During Smartphone Use **eTable 6.** Association of Smartphone Use Duration With Underperception and Overperception of Body Weight Among Male and Female Adolescents, Stratified by Body Mass Index **eTable 7.** Association of Smartphone Content Type With Body Image Distortion and Weight Loss Behaviors With Additional Adjustment for Body Image Distortion Among Male and Female Adolescents **eTable 8.** Association of Smartphone Use Duration With Body Image Distortion and Weight Loss Behaviors Among Male and Female Adolescents, Stratified by Household Income and Parental Educational Level **eTable 9.** Association of Weekday and Weekend Smartphone Use Duration With Body Image Distortion and Weight Loss Behaviors **eTable 10.** Association of Smartphone Use Duration With Body Image Distortion (Overperception of Body Weight) and Weight Loss Behaviors (Weight Loss Attempts, Use of Inappropriate Weight Loss Strategies, Muscle-Strengthening Activity, and Aerobic Physical Activity) in Male and Female Adolescents After Excluding Adolescents Who Were Overweight **eTable 11.** Association of Smartphone Content Types With Body Image Distortion (Overperception of Body Weight) and Weight Loss Behaviors (Weight Loss Attempts, Use of Inappropriate Weight Loss Strategies, Muscle-Strengthening Activity, and Aerobic Physical Activity) in Male and Female Adolescents After Excluding Adolescents Who Were Overweight **eTable 12.** Association of Smartphone Use Duration With Body Image Distortion (Overperception of Body Weight) and Weight Loss Behaviors (Weight Loss Attempts, Use of Inappropriate Weight Loss Strategies, Muscle-Strengthening Activity, and Aerobic Physical Activity) in Male and Female Adolescents in the KYRBS 2020 Data
