# Supplementary material for: Association of Smartphone Use With Body Image Distortion and Weight Loss Behaviors in Korean Adolescents
Source: JAMA Netw Open. 2022 May 20;5(5):e2213237. doi: 10.1001/jamanetworkopen.2022.13237 (PMC9123497; doi:10.1001/jamanetworkopen.2022.13237)
Supplement: Supplement. — eTable 1. Participant Characteristics According Smartphone Use Duration, Including Adolescents Who Reported No Smartphone Use During the Past 30 Days eTable 2. Distribution of Smartphone Use Duration (Minutes/Day) According to Individuals’ Most Frequently Used Smartphone Content Type in Male and Female Adolescents eTable 3. Association of Smartphone Use Duration With Body Image Distortion (Overperception of Body Weight) and Weight Loss Behaviors (Weight Loss Attempts, Use of Inappropriate Weight Loss Strategies, Muscle-Strengthening Activity, and Aerobic Physical Activity) With Additional Adjustment for Body Image Distortion Among Male and Female Adolescents eTable 4. Association of Smartphone Use Duration With Weight Loss Behaviors (Weight Loss Attempts, Use of Inappropriate Weight Loss Strategies, Muscle-Strengthening Activity, and Aerobic Physical Activity) in Male and Female Adolescents, Stratified by Body Image Distortion eTable 5. Association of Smartphone Use Duration With Body Image Distortion and Weight Loss Behaviors Among Male and Female Adolescents, Stratified by Types of Content Most Frequently Accessed During Smartphone Use eTable 6. Association of Smartphone Use Duration With Underperception and Overperception of Body Weight Among Male and Female Adolescents, Stratified by Body Mass Index eTable 7. Association of Smartphone Content Type With Body Image Distortion and Weight Loss Behaviors With Additional Adjustment for Body Image Distortion Among Male and Female Adolescents eTable 8. Association of Smartphone Use Duration With Body Image Distortion and Weight Loss Behaviors Among Male and Female Adolescents, Stratified by Household Income and Parental Educational Level eTable 9. Association of Weekday and Weekend Smartphone Use Duration With Body Image Distortion and Weight Loss Behaviors eTable 10. Association of Smartphone Use Duration With Body Image Distortion (Overperception of Body Weight) and Weight Loss Behaviors (Weight Loss Attempts, Use of [file jamanetwopen-e2213237-s001.pdf]

Supplementary Online Content

Kwon S, Kim R, Lee JT, et al. Association of smartphone use with body image distortion and weight loss behaviors in Korean adolescents. *JAMA Netw Open*. 2022;5(5):e2213237. doi:10.1001/jamanetworkopen.2022.13237

eTable 1. Participant Characteristics According Smartphone Use Duration, Including Adolescents Who Reported No Smartphone Use During the Past 30 Days

eTable 2. Distribution of Smartphone Use Duration (Minutes/Day) According to Individuals' Most Frequently Used Smartphone Content Type in Male and Female Adolescents

eTable 3. Association of Smartphone Use Duration With Body Image Distortion (Overperception of Body Weight) and Weight Loss Behaviors (Weight Loss Attempts, Use of Inappropriate Weight Loss Strategies, Muscle-Strengthening Activity, and Aerobic Physical Activity) With Additional Adjustment for Body Image Distortion Among Male and Female Adolescents

eTable 4. Association of Smartphone Use Duration With Weight Loss Behaviors (Weight Loss Attempts, Use of Inappropriate Weight Loss Strategies, Muscle-Strengthening Activity, and Aerobic Physical Activity) in Male and Female Adolescents, Stratified by Body Image Distortion

eTable 5. Association of Smartphone Use Duration With Body Image Distortion and Weight Loss Behaviors Among Male and Female Adolescents, Stratified by Types of Content Most Frequently Accessed During Smartphone Use

eTable 6. Association of Smartphone Use Duration With Underperception and Overperception of Body Weight Among Male and Female Adolescents, Stratified by Body Mass Index

eTable 7. Association of Smartphone Content Type With Body Image Distortion and Weight Loss Behaviors With Additional Adjustment for Body Image Distortion Among Male and Female Adolescents

eTable 8. Association of Smartphone Use Duration With Body Image Distortion and Weight Loss Behaviors Among Male and Female Adolescents, Stratified by Household Income and Parental Educational Level

eTable 9. Association of Weekday and Weekend Smartphone Use Duration With Body Image Distortion and Weight Loss Behaviors

eTable 10. Association of Smartphone Use Duration With Body Image Distortion (Overperception of Body Weight) and Weight Loss Behaviors (Weight Loss Attempts, Use of Inappropriate Weight Loss Strategies, Muscle-Strengthening Activity, and Aerobic Physical Activity) in Male and Female Adolescents After Excluding Adolescents Who Were Overweight

eTable 11. Association of Smartphone Content Types With Body Image Distortion (Overperception of Body Weight) and Weight Loss Behaviors (Weight Loss Attempts, Use of Inappropriate Weight Loss Strategies, Muscle-Strengthening Activity, and Aerobic Physical Activity) in Male and Female Adolescents After Excluding Adolescents Who Were Overweight

eTable 12. Association of Smartphone Use Duration With Body Image Distortion (Overperception of Body Weight) and Weight Loss Behaviors (Weight Loss Attempts, Use of Inappropriate Weight Loss Strategies, Muscle-Strengthening Activity, and Aerobic Physical Activity) in Male and Female Adolescents in the KYRBS 2020 Data

This supplementary material has been provided by the authors to give readers additional information about their work.

© 2022 Kwon S et al. *JAMA Network Open*.

eTable 1. Participant Characteristics According Smartphone Use Duration, Including Adolescents Who Reported No Smartphone Use During the Past 30 Days

		Duration of Smartphone use (min/d)				
		0	1-120	121-180	181-300	≥301
N		7,259	14,092	11,144	15,043	12,854
Weighted N [a]		351,129	698,144	551,804	730,726	605,491
		N (weighted % [a])				
School [b]	Middle School	3,721 (46.7)	6,994 (45.8)	5,358 (43.8)	7,462 (45.2)	6,527 (46.3)
	High School	3,538 (53.3)	7,098 (54.2)	5,786 (56.2)	7,581 (54.8)	6,327 (53.7)
Sex	Male	4,442 (62.9)	8,641 (62.1)	5,911 (54.4)	6,746 (45.9)	4,922 (39.6)
	Female	2,817 (37.1)	5,451 (37.9)	5,233 (45.6)	8,297 (54.1)	7,932 (60.4)
Household Income	Low	1,003 (13.3)	1,402 (9.8)	1,335 (11.8)	2,219 (14.6)	2,450 (19.0)
	Middle	3,178 (43.6)	5,802 (40.6)	5,256 (46.9)	7,395 (49.1)	6,289 (48.8)
	High	3,078 (43.1)	6,888 (49.6)	4,553 (41.3)	5,429 (36.4)	4,115 (32.2)
Parental Co-residence	Both Parents	5,916 (82.7)	12,325 (88.1)	9,585 (86.8)	12,504 (83.7)	9,997 (78.4)
	Single Parent	1,013 (13.1)	1,455 (10)	1,319 (11.4)	2,167 (14.0)	2,405 (18.3)
	Not living with parents	330 (4.2)	312 (1.9)	240 (1.8)	372 (2.3)	452 (3.3)
Parental Education [c]	Middle school or less	98 (1.2)	83 (0.5)	79 (0.7)	152 (0.9)	186 (1.3)
	High school	1,768 (23.9)	2,408 (16.8)	2,477 (22.2)	4,050 (26.6)	3,986 (30.7)
	College or more	3,917 (55.9)	10,071 (73.0)	7,190 (65.4)	8,736 (59.2)	6,267 (50.2)
	Missing	1,476 (19)	1,530 (9.7)	1,398 (11.7)	2,105 (13.3)	2,415 (17.8)
Region	Rural	614 (6.8)	946 (5.6)	717 (5.0)	1,159 (6.0)	1,221 (7.5)
	Small and Medium-sized urban	3,565 (52.2)	6,274 (46.9)	5,296 (50.5)	7,277 (51.5)	6,471 (53.8)
	Metropolitan urban	3,080 (41)	6,872 (47.6)	5,131 (44.6)	6,607 (42.5)	5,162 (38.8)
Perceived stress level	Low	1,763 (23.8)	3,432 (23.7)	2,336 (20.4)	2,799 (18.5)	2,038 (16.0)
	Moderate	3,013 (42.4)	6,065 (43.5)	5,011 (45.5)	6,470 (43.1)	5,094 (40.0)
	High	2,483 (33.9)	4,595 (32.8)	3,797 (34.1)	5,774 (38.4)	5,722 (44.0)
Presence of depressive Symptoms		1,614 (22.1)	2,857 (20.4)	2,492 (22.5)	3,851 (25.7)	4,138 (32.2)
Body mass index [d]	Underweight	524 (7.3)	982 (7.1)	743 (6.5)	1,054 (7)	863 (6.9)
	Normal weight	5,284 (72.8)	10,549 (74.8)	8,385 (75.6)	11,099 (73.9)	9272 (72.1)
	Overweight	1,451 (19.9)	2,561 (18.1)	2,016 (17.9)	2,890 (19.1)	2,719 (21.0)
Body image distortion [e]		1,728 (23.7)	3,116 (22.2)	2,703 (24.6)	4,067 (27)	3,731 (28.8)
Weight-loss attempt		2,336 (31.7)	3,837 (27)	3,444 (30.5)	5,277 (34.9)	5,061 (39.0)
Use of inappropriate weight-loss strategies		526 (7.2)	511 (3.5)	526 (4.6)	945 (6.2)	1,382 (10.5)
Muscle-strengthening activity	Meeting the guidelines	1,836 (25.4)	3,570 (25)	2,592 (22.9)	3,211 (21.2)	2,672 (20.6)
Aerobic physical activity	Meeting the guidelines	2,903 (39.3)	6,147 (42.2)	4,513 (39.3)	5,639 (36.4)	4,688 (35.7)

[a] Weighted N and weighted percentage reflect weighted counts when weighted back to the entire Korean middle and high school students.
[b] Grade in school was used as a proxy for age in the analyses.
[c] Organization for Economic Co-operation and Development (OECD) parental education indicators: middle school or lower indicates both parents completed middle school education or less; high school indicates at least 1 parent graduated from high school; college or higher indicates at least 1 parent graduated from college or more.
[d] Underweight was defined as BMI for age that was lower than the 5th percentile, normal weight as BMI for age that was between the 5th and 84th percentile, and overweight as BMI for age that was in the 85th percentile or higher based on the 2017 Korean National Growth Chart.
[e] Body image distortion was defined as overperception of body weight, which occurred when participants who were underweight perceived themselves as normal weight, fat, or very fat; when participants who were normal weight perceived themselves as fat or very fat; and when participants who were overweight perceived themselves as very fat.

© 2022 Kwon S et al. *JAMA Network Open*.

eTable 2. Distribution of Smartphone Use Duration (Minutes/Day) According to Individuals' Most Frequently Used Smartphone Content Type in Male and Female Adolescents[a]

	Content type of Smartphone use	Mean	SE [b]	Median	Q1 [c]	Q3 [c]	IQR [d]
**Male**	**Education/ Information search**	157.56	3.69	118.80	59.82	196.40	136.58
	**Chatting/Messenger/ E-mail**	228.32	3.43	177.47	108.82	279.67	170.85
	**SNS/forum**	248.19	4.39	187.98	119.61	299.15	179.54
	**Videos/Movies/Music**	212.41	2.66	171.04	110.67	256.83	146.16
	**Webtoons/ Web-novel**	196.12	4.01	154.16	94.03	246.07	152.04
	**Game**	207.84	2.56	166.74	97.85	256.55	158.7
	**Shopping/Others**	286.65	19.01	193.67	109.78	327.11	217.33
**Female**	**Education/ Information search**	159.59	4.46	135.86	68.19	203.90	135.71
	**Chatting/Messenger/ E-mail**	280.21	3.75	213.91	136.99	341.91	204.92
	**SNS/forum**	294.98	3.48	235.42	154.25	365.74	211.49
	**Videos/Movies/Music**	244.14	3.35	204.91	136.02	299.79	163.77
	**Webtoons/ Web-novel**	222.38	3.90	188.41	115.04	284.67	169.63
	**Games**	265.87	6.79	213.85	135.11	337.77	202.66
	**Shopping/Others**	259.95	12.88	210.80	120.58	331.93	211.35

[a] Analyses were weighted back to represent the entire Korean middle and high school students.
[b] SE=Standard Error
[c] Q=Quartile
[d] IQR=Interquartile range

© 2022 Kwon S et al. *JAMA Network Open*.

eTable 3. Association of Smartphone Use Duration With Body Image Distortion (Overperception of Body Weight) and Weight Loss Behaviors (Weight Loss Attempts, Use of Inappropriate Weight Loss Strategies, Muscle-Strengthening Activity, and Aerobic Physical Activity) With Additional Adjustment for Body Image Distortion Among Male and Female Adolescents

Outcomes	Sex stratum	Number of events	Odds ratio (95% confidence interval) [a] Duration of Smartphone use (min/day)				P-trend [b]	P-interaction by sex [c]
			1-120	121-180	181-300	≥301		
Weight-loss attempt	Male	6174	1.0 (Ref)	1.01 (0.92-1.10)	1.12 (1.04-1.22)	1.03 (0.93-1.13)	0.34	<0.001 [f]
	Female	11445	1.0 (Ref)	1.19 (1.11-1.29)	1.33 (1.24-1.43)	1.54 (1.43-1.66)	<0.001 [f]	
Use of inappropriate weight-loss strategies	Male	782	1.0 (Ref)	1.07 (0.85-1.34)	1.15 (0.95-1.40)	1.50 (1.22-1.85)	<0.001 [f]	<0.001 [f]
	Female	2582	1.0 (Ref)	1.22 (1.06-1.42)	1.52 (1.32-1.74)	2.41 (2.11-2.75)	<0.001 [f]	
Muscle-strengthening activity [d]	Male	9020	1.0 (Ref)	1.00 (0.93-1.08)	1.05 (0.98-1.12)	1.04 (0.96-1.12)	0.20	0.60
	Female	3025	1.0 (Ref)	0.96 (0.85-1.08)	0.94 (0.84-1.06)	1.05 (0.93-1.18)	0.21	
Aerobic physical activity [e]	Male	13870	1.0 (Ref)	1.03 (0.96-1.11)	1.01 (0.95-1.08)	1.05 (0.97-1.13)	0.32	0.93
	Female	7117	1.0 (Ref)	0.96 (0.88-1.05)	0.95 (0.87-1.04)	1.02 (0.93-1.11)	0.39	

[a] Adjusted for grade in school (continuous), sex (male or female), household income (low, middle, or high), parental coresidence (both parents, single parent, or not living with parents or others), parental education (middle school or less, high school, college or more, or missing), region (rural, small and medium-sized urban, or metropolitan urban), perceived stress level (low, moderate, or high), depressive symptoms (yes or no), BMI (underweight, normal weight, or overweight), type of content most frequently accessed during smartphone use (educational or informational searches; chatting, messaging, or emails; games; videos, movies, or music; webtoons or web novels; social networking services or forums; or shopping or other activities)., and body image distortion (with or without).

[b] *P* for trend was estimated using the Wald test for continuous duration of smartphone use (with category-specific medians).

[c] *P* for interaction by sex was estimated using the Wald test for interaction term between sex and continuous duration of smartphone use (category-specific medians).

[d] Defined as engagement in muscle-strengthening activity for at least 3 days per week.

[e] Defined as engagement in moderate- to vigorous-intensity aerobic physical at least 5 days per week or vigorous-intensity physical activity at least 3 days per week.

[f] Nominal statistical significance at Bonferroni-corrected α=.01 (5 tests per exposure).

© 2022 Kwon S et al. *JAMA Network Open*.

eTable 4. Association of Smartphone Use Duration With Weight Loss Behaviors (Weight Loss Attempts, Use of Inappropriate Weight Loss Strategies, Muscle-Strengthening Activity, and Aerobic Physical Activity) in Male and Female Adolescents, Stratified by Body Image Distortion

Outcomes	Body image distortion	Number of event/ N	Per 1-hour increment in duration OR (95 % CI) [a]	p-int [b]
**Male adolescents**				
**Weight-loss attempt**	**Absent**	3941/21015	1.01 (1.00-1.02)	<0.001
	**Present**	2233/5205	1.00 (0.98-1.01)	
**Use of inappropriate weight-loss strategies**	**Absent**	490/21015	1.06 (1.03-1.08)	<0.001
	**Present**	292/5205	1.06 (1.03-1.10)	
**Muscle-strengthening activity** [c]	**Absent**	7655/21015	1.01 (1.00-1.02)	<0.001
	**Present**	1365/5205	1.01 (0.99-1.03)	
**Aerobic physical activity** [d]	**Absent**	11402/21015	1.01 (1.00-1.01)	<0.001
	**Present**	2468/5205	1.01 (0.99-1.03)	
**Female adolescents**				
**Weight-loss attempt**	**Absent**	6820/18501	1.04 (1.03-1.05)	<0.001
	**Present**	4625/8412	1.04 (1.03-1.06)	
**Use of inappropriate weight-loss strategies**	**Absent**	1435/18501	1.09 (1.08-1.11)	<0.001
	**Present**	1147/8412	1.08 (1.06-1.09)	
**Muscle-strengthening activity** [c]	**Absent**	2174/18501	1.02 (1.00-1.03)	0.002
	**Present**	851/8412	1.00 (0.98-1.02)	
**Aerobic physical activity** [d]	**Absent**	4978/18501	1.01 (1.00-1.02)	0.73
	**Present**	2139/8412	1.01 (1.00-1.03)	

[b] Adjusted for grade in school (continuous), sex (male or female), household income (low, middle, or high), parental coresidence (both parents, single parent, or not living with parents or others), parental education (middle school or less, high school, college or more, or missing), region (rural, small and medium-sized urban, or metropolitan urban), perceived stress level (low, moderate, or high), depressive symptoms (yes or no), BMI (underweight, normal weight, or overweight), and type of content most frequently accessed during smartphone use (educational or informational searches; chatting, messaging, or emails; games; videos, movies, or music; webtoons or web novels; social networking services or forums; or shopping or other activities).

[b] *P* for interaction by body image distortion was estimated using the Wald test for interaction term between body image distortion and duration of smartphone use.

[c] Defined as engagement in muscle-strengthening activity for at least 3 days per week.

[d] Defined as engagement in moderate- to vigorous-intensity aerobic physical activity at least 5 days per week or vigorous-intensity physical activity at least 3 days per week.

© 2022 Kwon S et al. *JAMA Network Open*.

eTable 5. Association of Smartphone Use Duration With Body Image Distortion and Weight Loss Behaviors Among Male and Female Adolescents, Stratified by Types of Content Most Frequently Accessed During Smartphone Use

Outcomes	Sex stratum	OR (95% CI) per 1-hour increment in duration of smartphone use [a]						P-interaction [b]
		Education/ information search	Chatting/ Messenger/E-mail	SNS/Forum	Video/Movies/ Music	Webtoon/Web-novel	Game	
**Body image distortion**	Male	1.04 (1.01-1.08)	0.99 (0.97-1.01)	1.03 (1.00-1.06)	1.00 (0.98-1.02)	0.99 (0.96-1.03)	1.03 (1.01-1.06)	<0.001
	Female	1.06 (1.00-1.12)	1.02 (1.00-1.03)	1.02 (1.00-1.03)	1.02 (1.00-1.04)	0.99 (0.96-1.02)	1.06 (1.01-1.11)	0.06
**Weight-loss attempt**	Male	1.05 (1.01-1.09)	1.00 (0.98-1.02)	1.01 (0.98-1.03)	1.01 (0.99-1.03)	0.96 (0.93-1.00)	1.01 (0.99-1.04)	<0.001
	Female	1.03 (0.99-1.08)	1.04 (1.03-1.05)	1.05 (1.03-1.06)	1.03 (1.01-1.05)	1.06 (1.03-1.09)	1.05 (0.99-1.10)	<0.001
**Use of inappropriate weight-loss strategies**	Male	1.12 (1.05-1.19)	1.06 (1.02-1.10)	1.06 (1.00-1.12)	1.07 (1.02-1.11)	0.99 (0.91-1.07)	1.06 (1.01-1.12)	0.16
	Female	1.12 (1.06-1.18)	1.08 (1.06-1.09)	1.10 (1.07-1.12)	1.09 (1.06-1.12)	1.06 (1.01-1.11)	1.11 (1.04-1.20)	<0.001
**Muscle-strengthening activity** [c]	Male	1.00 (0.97-1.03)	1.01 (1.00-1.03)	1.01 (0.98-1.03)	1.01 (0.99-1.02)	1.00 (0.96-1.04)	1.00 (0.98-1.01)	<0.001
	Female	1.01 (0.95-1.08)	1.01 (0.99-1.03)	1.01 (0.99-1.03)	1.01 (0.98-1.04)	0.97 (0.92-1.03)	1.01 (0.92-1.10)	0.002
**Aerobic physical activity** [d]	Male	1.01 (0.99-1.05)	1.00 (0.98-1.01)	1.01 (0.99-1.03)	1.00 (0.98-1.02)	1.02 (0.98-1.05)	1.01 (0.99-1.03)	<0.001
	Female	1.04 (0.99-1.09)	1.04 (0.99-1.09)	1.03 (1.01-1.04)	1.00 (0.98-1.03)	0.99 (0.96-1.03)	1.00 (0.95-1.06)	0.03

[a] Adjusted for grade in school (continuous), sex (male or female), household income (low, middle, or high), parental coresidence (both parents, single parent, or not living with parents or others), parental education (middle school or less, high school, college or more, or missing), region (rural, small and medium-sized urban, or metropolitan urban), perceived stress level (low, moderate, or high), depressive symptoms (yes or no), and BMI (underweight, normal weight, or overweight).
[b] *P* for interaction by types of content most frequently accessed during smartphone use was estimated using a global *F* test for interaction term between types of content accessed during smartphone use and continuous duration of smartphone use.
[c] Defined as engagement in muscle-strengthening activity for at least 3 days per week.
[d] Defined as engagement in moderate to vigorous aerobic physical activity at least 5 days per week or vigorous physical activity at least 3 days per week.

© 2022 Kwon S et al. *JAMA Network Open*.

eTable 6. Association of Smartphone Use Duration With Underperception and Overperception of Body Weight Among Male and Female Adolescents, Stratified by Body Mass Index

BMI stratum [a]	Sex stratum	Body image distortion outcome categories	Odds ratio (95% confidence interval)				P-trend [b]
			Duration of Smartphone use (min/day)				
			1-120	121-180	181-300	≥301	
Underweight [c]	Male	Over-perception [d]	1.0 (Ref)	0.71 (0.43-1.16)	0.82 (0.45-1.47)	1.00 (0.42-2.40)	0.06
	Female	Over-perception [d]	1.0 (Ref)	0.59 (0.38-0.91)	0.86 (0.57-1.31)	0.82 (0.46-1.46)	0.11
Normal weight [e]	Male	Under-perception [f]	1.0 (Ref)	1.11 (1.02-1.21)	1.12 (1.03-1.23)	1.18 (1.07-1.30)	0.002
		Over-perception [f]	1.0 (Ref)	1.10 (1.00-1.22)	1.11 (1.00-1.23)	1.11 (0.99-1.26)	0.09
	Female	Under-perception [f]	1.0 (Ref)	1.01 (0.90-1.13)	1.00 (0.89-1.11)	0.94 (0.84-1.05)	0.18
		Over-perception [f]	1.0 (Ref)	1.12 (1.02-1.22)	1.18 (1.08-1.30)	1.18 (1.07-1.30)	0.005
Overweight [g]	Male	Under-perception [f]	1.0 (Ref)	0.94 (0.71-1.24)	0.75 (0.55-1.01)	1.01 (0.76-1.36)	0.90
		Over-perception [f]	1.0 (Ref)	1.16 (0.95-1.41)	1.33 (1.12-1.58)	1.44 (1.20-1.73)	<0.001
	Female	Under-perception [f]	1.0 (Ref)	1.12 (0.68-1.83)	1.15 (0.73-1.81)	1.04 (0.64-1.70)	0.95
		Over-perception [f]	1.0 (Ref)	1.00 (0.80-1.27)	1.12 (0.90-1.39)	1.34 (1.07-1.67)	0.002

[a] Underweight was defined as BMI for age that was lower than the 5th percentile, normal weight as BMI for age that was between 5th and 84th percentile, and overweight as BMI for age that was in the 85th percentile based on the 2017 Korean national growth chart.

[b] *P* for trend was estimated using the Wald test for continuous duration of smartphone use (with category-specific medians).

[c] Among underweight adolescents, over-perception of body weight was defined as those perceived themselves as normal weight, fat, or very fat.

[d] Among underweight adolescents, OR and 95% CI were estimated using binary logistic regression models (over-perception vs. accurate perception), adjusting for grade in school (continuous), sex (male or female), household income (low, middle, or high), parental co-residence (both parents, single parent, or not living with parents or others), parental education (middle school or less, high school, college or more, or missing), region (rural, small and medium-sized urban, or metropolitan urban), perceived stress level (low, moderate, or high), depressive symptoms (yes or no), and type of content most frequently accessed during smartphone use (educational or informational searches; chatting, messaging, or emails; games; videos, movies, or music; webtoons or web novels; social networking services or forums; or shopping or other activities).

[e] Among normal weight adolescents, under-perception of body weight was defined as those perceived themselves as very lean or lean, and over-perception of body weight as those perceived themselves as fat or very fat.

[f] Among normal weight and overweight adolescents, OR and 95% CI were estimated using polytomous logistic regression models (under-perception, over-perception vs. accurate perception), adjusting for grade in school (continuous), sex (male or female), household income (low, middle, or high), parental coresidence (both parents, single parent, or not living with parents or others), parental education (middle school or less, high school, college or more, or missing), region (rural, small and medium-sized urban, or metropolitan urban), perceived stress level (low, moderate, or high), depressive symptoms (yes or no), and type of content most frequently accessed during smartphone use (educational or informational searches; chatting, messaging, or emails; games; videos, movies, or music; webtoons or web novels; social networking services or forums; or shopping or other activities).

[g] Among overweight adolescents, under-perception of body weight was defined as those perceived themselves as very lean, lean, or normal weight, and over-perception of body weight as those perceived themselves as very fat.

© 2022 Kwon S et al. *JAMA Network Open*.

eTable 7. Association of Smartphone Content Type With Body Image Distortion and Weight Loss Behaviors With Additional Adjustment for Body Image Distortion Among Male and Female Adolescents

Outcomes		Number of event	Odds ratio (95% confidence interval) [a]							P-value [b]	P-interaction by sex [c]
			Content type of smartphone use								
			Education/ Information search	Chatting/ Messenger/ E-mail	SNS/ Forum	Video/ Movies/ Music	Webtoon/ Web-novel	Game	Shopping/ Others		
Weight-loss attempt	Male	6,174	1.0 (Ref)	1.12 (0.98-1.27)	1.17 (1.02-1.34)	1.04 (0.92-1.17)	0.84 (0.72-0.98)	0.90 (0.80-1.02)	0.81 (0.58-1.14)	<0.001 [f]	<0.001 [f]
	Female	11,445	1.0 (Ref)	1.35 (1.20-1.52)	1.21 (1.07-1.37)	1.07 (0.94-1.21)	0.85 (0.75-0.98)	0.70 (0.58-0.83)	1.27 (0.99-1.62)	<0.001 [f]	
Use of inappropriate weight-loss strategies	Male	782	1.0 (Ref)	1.06 (0.76-1.47)	1.21 (0.86-1.70)	0.97 (0.71-1.32)	0.74 (0.51-1.08)	0.87 (0.63-1.21)	1.01 (0.50-2.05)	0.04	0.05
	Female	2,582	1.0 (Ref)	1.58 (1.25-2.00)	1.36 (1.08-1.73)	1.12 (0.88-1.44)	0.91 (0.69-1.20)	0.93 (0.65-1.33)	1.36 (0.89-2.08)	<0.001 [f]	
Muscle-strengthening activity [d]	Male	9,020	1.0 (Ref)	1.30 (1.18-1.44)	1.26 (1.12-1.41)	0.96 (0.87-1.05)	0.97 (0.86-1.10)	0.83 (0.75-0.92)	1.42 (1.08-1.86)	<0.001 [f]	<0.001 [f]
	Female	3,025	1.0 (Ref)	1.08 (0.89-1.31)	0.96 (0.79-1.17)	1.10 (0.91-1.33)	0.82 (0.66-1.02)	0.77 (0.58-1.02)	1.00 (0.68-1.47)	<0.001 [f]	
Aerobic physical activity [e]	Male	13,870	1.0 (Ref)	1.41 (1.28-1.55)	1.28 (1.15-1.42)	1.01 (0.92-1.12)	0.97 (0.86-1.10)	0.87 (0.79-0.96)	1.82 (1.40-2.37)	<0.001 [f]	<0.001 [f]
	Female	7,117	1.0 (Ref)	0.95 (0.84-1.08)	0.85 (0.74-0.97)	0.97 (0.84-1.11)	0.90 (0.78-1.04)	0.72 (0.59-0.89)	1.05 (0.78-1.41)	<0.001 [f]	

[a] Adjusted for grade in school (continuous), sex (male or female), household income (low, middle, or high), parental coresidence (both parents, single parent, or not living with parents or others), parental education (middle school or less, high school, college or more, or missing), region (rural, small and medium-sized urban, or metropolitan urban), perceived stress level (low, moderate, or high), depressive symptoms (yes or no), BMI (underweight, normal weight, or overweight), duration of smartphone use (≤120 minutes/day, 121-180 minutes/day, 181-300 minutes/day, or ≥301 minutes/day), and body image distortion (with or without).

[b] *P* value was estimated using a global *F* test comparing across types of content most frequently accessed during smartphone use.

[c] *P* for interaction by sex was estimated using a global *F* test for interaction terms between sex and types of content accessed during smartphone use.

[d] Defined as engagement in muscle-strengthening activity for at least 3 days per week.

[e] Defined as engagement in moderate- to vigorous-intensity aerobic physical activity at least 5 days per week or vigorous-intensity physical activity at least 3 days per week.

[f] Nominal statistical significance at Bonferroni-corrected α=.0125 (4 tests per exposure)

© 2022 Kwon S et al. *JAMA Network Open*.

eTable 8. Association of Smartphone Use Duration With Body Image Distortion and Weight Loss Behaviors Among Male and Female Adolescents, Stratified by Household Income and Parental Educational Level

			OR (95% CI) per 1-hour increment in duration of smartphone use [a]				
			Outcomes				
			Body image distortion	Weight-loss attempt	Use of inappropriate weight-loss strategies	Muscle-strengthening activity [b]	Aerobic physical activity [c]
**Household income**	**Male**	**Low**	1.01 (0.99-1.03)	1.02 (0.99-1.04)	1.09 (1.04-1.13)	0.99 (0.97-1.02)	1.01 (0.99-1.03)
		**Middle**	1.01 (1.00-1.03)	1.00 (0.98-1.01)	1.07 (1.03-1.10)	1.00 (0.98-1.01)	1.00 (0.98-1.01)
		**High**	1.01 (1.00-1.03)	1.01 (0.99-1.02)	1.05 (1.01-1.08)	1.02 (1.01-1.04)	1.01 (1.00-1.02)
		**p-interaction [d]**	0.96	0.35	0.35	0.01	0.20
	**Female**	**Low**	1.01 (0.99-1.02)	1.03 (1.01-1.04)	1.05 (1.03-1.08)	0.98 (0.95-1.01)	1.00 (0.98-1.03)
		**Middle**	1.01 (1.00-1.02)	1.04 (1.03-1.05)	1.08 (1.06-1.10)	1.00 (0.99-1.02)	1.01 (0.99-1.02)
		**High**	1.03 (1.02-1.04)	1.06 (1.04-1.07)	1.11 (1.09-1.13)	1.04 (1.02-1.06)	1.02 (1.00-1.03)
		**p-interaction [d]**	0.04	0.01	<0.001	0.01	0.51
**Parental education**	**Male**	**Middle school or less**	1.04 (0.92-1.17)	1.13 (1.02-1.25)	1.13 (0.99-1.29)	1.01 (0.93-1.09)	1.01 (0.94-1.09)
		**High school**	1.00 (0.98-1.02)	1.00 (0.98-1.02)	1.04 (1.00-1.08)	1.00 (0.99-1.02)	1.00 (0.99-1.02)
		**College or more**	1.02 (1.01-1.04)	1.01 (1.00-1.03)	1.07 (1.04-1.10)	1.01 (1.00-1.02)	1.01 (0.99-1.02)
		**p-interaction [e]**	0.12	0.82	0.87	0.44	0.72
	**Female**	**Middle school or less**	0.99 (0.93-1.05)	1.05 (0.98-1.12)	1.08 (0.99-1.18)	1.04 (0.94-1.15)	1.06 (0.98-1.14)
		**High school**	1.01 (1.00-1.03)	1.04 (1.02-1.06)	1.08 (1.06-1.10)	1.02 (1.00-1.04)	1.02 (1.00-1.03)
		**College or more**	1.02 (1.01-1.03)	1.05 (1.04-1.06)	1.10 (1.09-1.12)	1.02 (1.01-1.04)	1.01 (1.00-1.02)
		**p-interaction [e]**	0.04	0.14	0.05	0.76	0.22

[a] Adjusted for grade in school (continuous), sex (male or female), household income (low, middle, or high), parental coresidence (both parents, single parent, or not living with parents or others), parental education (middle school or less, high school, college or more, or missing), region (rural, small and medium-sized urban, or metropolitan urban), perceived stress level (low, moderate, or high), depressive symptoms (yes or no), BMI (underweight, normal weight, or overweight), and type of content most frequently accessed during smartphone use (educational or informational searches; chatting, messaging, or emails; games; videos, movies, or music; webtoons or web novels; social networking services or forums; or shopping or other activities).

[b] Defined as engagement in muscle-strengthening activity for at least 3 days per week.

[c] Defined as engagement in moderate- to vigorous-intensity aerobic physical activity at least 5 days per week or vigorous-intensity physical activity at least 3 days per week.

[d] *P* for interaction by household income was estimated using a global *F* test for interaction term between household income and duration of smartphone use.

[e] *P* for interaction by parental education was estimated using a global *F* test for interaction term between parental education and duration of smartphone use.

© 2022 Kwon S et al. *JAMA Network Open*.

eTable 9. Association of Weekday and Weekend Smartphone Use Duration With Body Image Distortion and Weight Loss Behaviors

Outcomes		OR (95 % CI) per 1-hour increment in duration of smartphone use	
		Male adolescents	Female adolescents
**Body image distortion**	**Weekday use [a]**	0.99 (0.98-1.01)	0.99 (0.98-1.00)
	**Weekend use [b]**	1.02 (1.01-1.03)	1.03 (1.02-1.04)
**Weight-loss attempt**	**Weekday use [a]**	0.99 (0.98-1.01)	1.03 (1.02-1.05)
	**Weekend use [b]**	1.01 (1.00-1.02)	1.01 (1.00-1.02)
**Use of inappropriate weight-loss strategies**	**Weekday use [a]**	1.04 (1.02-1.07)	1.06 (1.04-1.08)
	**Weekend use [b]**	1.02 (0.99-1.04)	1.02 (1.01-1.04)
**Muscle-strengthening activity [c]**	**Weekday use [a]**	0.99 (0.98-1.01)	1.03 (1.01-1.04)
	**Weekend use [b]**	1.01 (1.00-1.02)	0.99 (0.97-1.00)
**Aerobic physical activity [d]**	**Weekday use [a]**	1.01 (1.00-1.02)	1.03 (1.01-1.04)
	**Weekend use [b]**	1.00 (0.99-1.01)	0.99 (0.98-1.00)

[a] Adjusted for grade in school (continuous), sex (male or female), household income (low, middle, or high), parental coresidence (both parents, single parent, or not living with parents or others), parental education (middle school or less, high school, college or more, or missing), region (rural, small and medium-sized urban, or metropolitan urban), perceived stress level (low, moderate, or high), depressive symptoms (yes or no), BMI (underweight, normal weight, or overweight), type of content most frequently accessed during smartphone use (educational or informational searches; chatting, messaging, or emails; games; videos, movies, or music; webtoons or web novels; social networking services or forums; or shopping or other activities), and duration of weekends smartphone use (hours/day)

[b] Adjusted for grade in school (continuous), sex (male or female), household income (low, middle, or high), parental coresidence (both parents, single parent, or not living with parents or others), parental education (middle school or less, high school, college or more, or missing), region (rural, small and medium-sized urban, or metropolitan urban), perceived stress level (low, moderate, or high), depressive symptoms (yes or no), BMI (underweight, normal weight, or overweight), type of content most frequently accessed during smartphone use (educational or informational searches; chatting, messaging, or emails; games; videos, movies, or music; webtoons or web novels; social networking services or forums; or shopping or other activities), and duration of weekdays smartphone use (hours/day)

[c] Defined as engagement in muscle-strengthening activity for at least 3 days per week.

[d] Defined as engagement in moderate- to vigorous-intensity aerobic physical activity at least 5 days per week or vigorous-intensity physical activity at least 3 days per week.

© 2022 Kwon S et al. *JAMA Network Open*.

eTable 10. Association of Smartphone Use Duration With Body Image Distortion (Overperception of Body Weight) and Weight Loss Behaviors (Weight Loss Attempts, Use of Inappropriate Weight Loss Strategies, Muscle-Strengthening Activity, and Aerobic Physical Activity) in Male and Female Adolescents After Excluding Adolescents Who Were Overweight

Outcomes		Number of events	Odds ratio (95% confidence interval) [a] Duration of Smartphone use (min/day)				P-trend [b]	P-interaction [c]
			1-120	121-180	181-300	≥301		
**Body image distortion [d]**	**Male**	3,920	1.0 (Ref)	1.05 (0.96-1.14)	1.06 (0.96-1.17)	1.08 (0.97-1.20)	0.17	0.01 [g]
	**Female**	7,234	1.0 (Ref)	1.08 (0.99-1.17)	1.16 (1.07-1.26)	1.17 (1.07-1.28)	0.001 [g]	
**Weight-loss attempt**	**Male**	3,282	1.0 (Ref)	1.04 (0.94-1.15)	1.16 (1.05-1.28)	1.08 (0.97-1.22)	0.09	<0.001 [g]
	**Female**	8,810	1.0 (Ref)	1.19 (1.10-1.30)	1.35 (1.25-1.46)	1.58 (1.45-1.72)	<0.001 [g]	
**Use of inappropriate weight-loss strategies**	**Male**	417	1.0 (Ref)	1.22 (0.90-1.64)	1.31 (0.99-1.73)	1.83 (1.37-2.46)	<0.001 [g]	0.01 [g]
	**Female**	1,951	1.0 (Ref)	1.23 (1.03-1.47)	1.63 (1.39-1.91)	2.67 (2.28-3.13)	<0.001 [g]	
**Muscle-strengthening activity [e]**	**Male**	7,387	1.0 (Ref)	0.99 (0.91-1.07)	1.06 (0.98-1.14)	1.07 (0.98-1.16)	0.07	0.68
	**Female**	2,539	1.0 (Ref)	0.99 (0.87-1.13)	0.94 (0.83-1.06)	1.05 (0.92-1.20)	0.34	
**Aerobic physical activity [f]**	**Male**	10,956	1.0 (Ref)	1.00 (0.92-1.08)	1.03 (0.96-1.10)	1.04 (0.95-1.13)	0.32	0.99
	**Female**	5,877	1.0 (Ref)	0.94 (0.86-1.04)	0.95 (0.86-1.04)	1.02 (0.93-1.13)	0.29	

[a] Adjusted for grade in school (continuous), sex (male or female), household income (low, middle, or high), parental coresidence (both parents, single parent, or not living with parents or others), parental education (middle school or less, high school, college or more, or missing), region (rural, small and medium-sized urban, or metropolitan urban), perceived stress level (low, moderate, or high), depressive symptoms (yes or no), BMI (underweight, normal weight, or overweight), and type of content most frequently accessed during smartphone use (educational or informational searches; chatting, messaging, or emails; games; videos, movies, or music; webtoons or web novels; social networking services or forums; or shopping or other activities).

[b] *P* for trend was estimated using the Wald test for continuous duration of smartphone use (with category-specific medians).

[c] *P* for interaction by sex was estimated using the Wald test for interaction term between sex and continuous duration of smartphone use (with category-specific medians).

[d] Body image distortion was defined as overperception of body weight, which occurred when participants who were underweight perceived themselves as normal weight, fat, or very fat; and when participants who were normal weight perceived themselves as fat or very fat.

[e] Defined as engagement in muscle-strengthening activity for at least 3 days per week.

[f] Defined as engagement in moderate- to vigorous-intensity aerobic physical activity at least 5 days per week or vigorous-intensity physical activity at least 3 days per week.

[g] Nominal statistical significance at Bonferroni-corrected α=.01 (5 tests per exposure).

© 2022 Kwon S et al. *JAMA Network Open*.

eTable 11. Association of Smartphone Content Types With Body Image Distortion (Overperception of Body Weight) and Weight Loss Behaviors (Weight Loss Attempts, Use of Inappropriate Weight Loss Strategies, Muscle-Strengthening Activity, and Aerobic Physical Activity) in Male and Female Adolescents After Excluding Adolescents Who Were Overweight

Outcomes		Number of event	Odds ratio (95% confidence interval) [a]							P-value [b]	P-interaction [c]
			Content type of smartphone use								
			Education/ Information search	Chatting/ Messenger/ E-mail	SNS/ Forum	Video/ Movies/ Music	Webtoon/ Web-novel	Game	Shopping/ Others		
Body image distortion [d]	Male	3,920	1.0 (Ref)	0.98 (0.86-1.11)	0.88 (0.75-1.03)	1.20 (1.05-1.37)	1.28 (1.08-1.52)	1.22 (1.06-1.41)	1.07 (0.73-1.58)	<0.001[g]	0.04
	Female	7,234	1.0 (Ref)	0.95 (0.83-1.10)	0.97 (0.84-1.12)	1.08 (0.93-1.24)	1.15 (0.98-1.35)	1.08 (0.87-1.33)	1.07 (0.79-1.44)	0.002 [g]	
Weight-loss attempt	Male	3,282	1.0 (Ref)	1.05 (0.91-1.21)	1.04 (0.88-1.22)	1.07 (0.93-1.23)	1.00 (0.83-1.20)	0.88 (0.76-1.03)	0.77 (0.5-1.19)	0.02	<0.001 [g]
	Female	8,810	1.0 (Ref)	1.30 (1.14-1.48)	1.18 (1.04-1.35)	1.06 (0.93-1.22)	0.85 (0.74-0.99)	0.71 (0.58-0.86)	1.27 (0.97-1.68)	<0.001 [g]	
Use of inappropriate weight-loss strategies	Male	417	1.0 (Ref)	0.97 (0.65-1.44)	1.13 (0.73-1.75)	0.97 (0.66-1.42)	0.59 (0.35-1.00)	0.76 (0.51-1.14)	1.07 (0.43-2.67)	0.06	0.22
	Female	1,951	1.0 (Ref)	1.46 (1.13-1.90)	1.27 (0.97-1.66)	1.08 (0.82-1.42)	0.93 (0.68-1.26)	0.88 (0.57-1.34)	1.23 (0.75-2.01)	<0.001 [g]	
Muscle-strengthening activity [e]	Male	7,387	1.0 (Ref)	1.32 (1.20-1.46)	1.31 (1.16-1.48)	0.94 (0.85-1.05)	1.02 (0.89-1.16)	0.83 (0.75-0.93)	1.51 (1.12-2.04)	<0.001 [g]	<0.001 [g]
	Female	2,539	1.0 (Ref)	1.04 (0.85-1.27)	0.96 (0.78-1.18)	1.12 (0.91-1.38)	0.80 (0.63-1.01)	0.69 (0.50-0.94)	1.01 (0.66-1.53)	<0.001 [g]	
Aerobic physical activity [f]	Male	10,956	1.0 (Ref)	1.35 (1.21-1.50)	1.22 (1.08-1.38)	0.98 (0.88-1.09)	0.96 (0.84-1.10)	0.86 (0.77-0.95)	1.72 (1.30-2.28)	<0.001 [g]	<0.001 [g]
	Female	5,877	1.0 (Ref)	0.91 (0.79-1.05)	0.82 (0.71-0.94)	0.99 (0.85-1.15)	0.91 (0.78-1.07)	0.74 (0.59-0.92)	0.93 (0.67-1.30)	0.001 [g]	

[a] Adjusted for grade in school (continuous), sex (male or female), household income (low, middle, or high), parental coresidence (both parents, single parent, or not living with parents or others), parental education (middle school or less, high school, college or more, or missing), region (rural, small and medium-sized urban, or metropolitan urban), perceived stress level (low, moderate, or high), depressive symptoms (yes or no), BMI (underweight, normal weight, or overweight), and duration of smartphone use (≤120 minutes/day, 121-180 minutes/day, 181-300 minutes/day, or≥301 minutes/day).

[b] *P* value was estimated using a global *F* test comparing across types of content most frequently accessed during smartphone use.

[c] *P* for interaction by sex was estimated using the global *F* test for interaction term between sex and types of content accessed during smartphone use.

[d] Body image distortion was defined as overperception of body weight, which occurred when participants who were underweight perceived themselves as normal weight, fat, or very fat; and when participants who were normal weight perceived themselves as fat or very fat.

[e] Defined as engagement in muscle-strengthening activity for at least 3 days per week.

[f] Defined as engagement in moderate- to vigorous-intensity aerobic physical activity at least 5 days per week or vigorous-intensity physical activity at least 3 days per week. [g] Nominal statistical significance at Bonferroni-corrected α=.01 (5 tests per exposure).

© 2022 Kwon S et al. *JAMA Network Open*.

eTable 12. Association of Smartphone Use Duration With Body Image Distortion (Overperception of Body Weight) and Weight Loss Behaviors (Weight Loss Attempts, Use of Inappropriate Weight Loss Strategies, Muscle-Strengthening Activity, and Aerobic Physical Activity) in Male and Female Adolescents in the KYRBS 2020 Data

Outcomes		Number of events	Odds ratio (95% confidence interval) [a]				P-trend [b]	P-interaction [c]
			Duration of Smartphone use (min/day)					
			1-120	121-180	181-300	≥301		
Body image distortion [d]	Male	3983	1.0 (Ref)	1.09 (0.97-1.22)	1.12 (1.00-1.26)	1.28 (1.15-1.42)	<0.001 [g]	0.72
	Female	5950	1.0 (Ref)	0.99 (0.85-1.16)	1.08 (0.95-1.22)	1.25 (1.10-1.41)	<0.001 [g]	
Weight-loss attempt	Male	5591	1.0 (Ref)	1.05 (0.92-1.19)	1.03 (0.93-1.15)	1.10 (0.98-1.22)	0.08	<0.001
	Female	9263	1.0 (Ref)	1.23 (1.06-1.41)	1.30 (1.16-1.46)	1.56 (1.40-1.74)	<0.001 [g]	
Muscle-strengthening activity [e]	Male	7417	1.0 (Ref)	1.06 (0.96-1.17)	1.13 (1.04-1.24)	1.19 (1.09-1.30)	<0.001 [g]	<0.001
	Female	2304	1.0 (Ref)	0.93 (0.76-1.13)	0.85 (0.71-1.01)	0.85 (0.71-1.00)	0.10	
Aerobic physical activity [f]	Male	8218	1.0 (Ref)	0.97 (0.88-1.06)	1.08 (0.99-1.18)	1.10 (0.99-1.21)	0.02	0.001
	Female	4125	1.0 (Ref)	1.07 (0.91-1.24)	1.00 (0.88-1.14)	0.95 (0.83-1.09)	0.07	

Note: Use of inappropriate weight-loss strategies information was not collected in the KYRBS 2020

[a] Adjusted for grade in school (continuous), sex (male or female), household income (low, middle, or high), parental coresidence (both parents, single parent, or not living with parents or others), parental education (middle school or less, high school, college or more, or missing), region (rural, small and medium-sized urban, or metropolitan urban), perceived stress level (low, moderate, or high), depressive symptoms (yes or no), and BMI (underweight, normal weight, or overweight).

[b] *P* for trend was estimated using the Wald test for continuous duration of smartphone use (with category-specific medians).

[c] *P* for interaction by sex was estimated using the Wald test for interaction term of sex and continuous duration of smartphone use (with category-specific median).

[d] Body image distortion was defined as overperception of body weight, which occurred when participants who were underweight perceived themselves as normal weight, fat, or very fat; when participants who were normal weight perceived themselves as fat or very fat; and when participants who were overweight perceived themselves as very fat.

[e] Defined as engagement in muscle-strengthening activity for at least 3 days per week.

[f] Defined as engagement in moderate- to vigorous-intensity aerobic physical activity at least 5 days per week or vigorous-intensity physical activity at least 3 days per week.

[g] Nominal statistical significance at Bonferroni-corrected α= .0125 (4 tests per exposure).

© 2022 Kwon S et al. *JAMA Network Open*.
